# Determinants of Anemia among women in Uganda: further analysis of the Uganda demographic and health surveys

**DOI:** 10.1186/s12889-019-8114-1

**Published:** 2019-12-30

**Authors:** Olivia Nankinga, Danstan Aguta

**Affiliations:** 1 Department of Population Studies, School of Statistics and Planning, College of Business and Management Sciences, Makerere University, Kampala, Uganda; 2 Uganda Bureau of Statistics, Statistics House, Kampala, Uganda

**Keywords:** Anemia prevalence, Women of reproductive age, Anemia determinants, Uganda

## Abstract

**Background:**

Anemia is a public health problem in many developing countries. It affects a sizable proportion of women of reproductive age. Anemia increases the risk of morbidity and mortality from infectious diseases, and can lead to poor fetal outcomes, and low productivity. This study examined the trends and determinants of anemia among women of reproductive age in Uganda.

**Methods:**

This study analyzed data from the Uganda Demographic and Health Surveys conducted in 2006, 2011, and 2016. The study was based on 10,956 weighted cases of women age 15–49. Bivariate analysis and multiple logistic regression analysis examined the association between the outcome variable and the determinants. Potential determinants of anemia in women were selected based on literature.

**Results:**

The results of the analysis show that anemia decreased in Uganda between 2006 and 2016, but with an increase between 2011 and 2016. The overall prevalence of anemia among women was 50, 23, and 32% respectively in 2006, 2011, and 2016. Women who were pregnant at the time of the survey had higher odds of being anemic across the surveys (OR 2.00, 95% CI 1.49–2.67; OR 1.47, 95% CI 1.02–2.10; OR 1.33, 95% CI 1.07–1.65). Women in households with nonimproved sources of drinking water also had higher odds for anemia (OR 1.32, 95% CI 1.09–1.61) in 2016. Wealth index, region and age were also significantly associated with anemia in women.

**Conclusion:**

In order to reduce anemia in women, there is need to target pregnant women during antenatal and postpartum visits, and ensure that nutrition education during such visits is supported. There is also need to ensure sustainable household access to safe water. This should be combined with interventions aimed at enhancing household wealth.

## Background

Anemia is a major public health concern [1], affecting approximately 1.6 billion people globally [2]. About one in every three women of reproductive age 15–49 suffers from anemia globally [3, 4]. In Africa, anemia affects 35% of women of reproductive age [4]. It also contributes significantly to the global disability burden.

Anemia is caused by many factors, including iron deficiency, infections, genetics, and other nutritional deficiencies [3–7]. The effects of anemia in a population are both short and long term. In pregnant women, anemia is related to poor maternal health and fetal outcomes, which may include infections, illnesses, and possible death for both the mother and the baby [4, 8].

Reported risk factors for anemia in women include residence, socioeconomic status, maternal education, and pregnancy status [5, 9, 10]. Women of reproductive age have higher risk of anemia due to regular blood loss with menstruation, pregnancy, and childbirth [4]. If the dietary requirements during pregnancy are not met or no iron supplements are taken, the risk of anemia among this group increases. In a number of households in developing countries, iron-rich foods needed to replenish such blood loss in women are either missing or insufficient [11].

Many countries have sought support to combat this preventable public health concern. In 2014, a comprehensive plan on maternal, infant, and young child nutrition was approved by the World Health Assembly, with anemia being one of the global targets for reduction (by 50%) by 2025 and subsequent alleviation [3, 12]. Furthermore, countries including India, Pakistan, Ethiopia, Yemen, Nigeria, Malawi, and Uganda have systematically established investigations to understand the problems in their local contexts in order to design targeted approaches to combat the condition [10, 13–15].

In Uganda the Ministry of Health has put in place deliberate interventions to improve maternal and child health outcomes through the Reproductive, Maternal, Neonatal and Child Health Sharpened Plan [16]. In addition, with support from development partners, the government embarked on a number of multisectoral nutrition interventions that include: the production of biofortified and iron-rich crops, provision of iron, anthelmintic and Vitamin A supplementation, promotion of breastfeeding, complementary feeding, family planning, delayed cord clamping, intensified malaria prevention and treatment, promotion of hygiene through increased access to water, improved latrines, hand washing, and infectious disease prevention [17–19].

Despite multisectoral efforts to reduce the burden of anemia in Uganda, the 2016 Uganda Demographic and Health Survey (UDHS) reported that the prevalence of anemia was 53% in children age 6–59 months, up from 49% in 2011, and was 32% in women of reproductive age, up from 23% in 2011 [20, 21].

Past studies in Uganda have examined anemia using survey data for specific districts or regions [10, 22–24], but few have used multiple DHS datasets or considered a country-wide anemia assessment. In contrast, our study addresses the above gap by analyzing the anemia situation at a national scale and considering nationally representative DHS data for three survey years (2006, 2011, and 2016). The analyses were conducted for women of reproductive age 15–49 years. The specific objective of this study was to identify factors associated with anemia in women in Uganda. We hypothesized that women in rural areas, poorer households, and with no education are more likely to be anemic. We further hypothesized that women who were not involved in decision making at household level would have a higher risk of anemia than those involved in decision making.

## Methods

### Data source

The study used datasets from the 2006, 2011, and 2016 Uganda Demographic and Health Survey (UDHS). The UDHS is a nationally representative population-based household survey, conducted every 5 years. The UDHS uses a stratified two-stage cluster sampling procedure. In the first stage, clusters are selected from sampling frames using the most recent census. Households are selected from each cluster at the second stage. The UDHS captures information in such areas as births to women age 15–49, women’s demographic and socioeconomic characteristics, household characteristics, maternal and child health and nutrition, access to health facilities and involvement in household decision making using questionnaires. It further includes testing of height and weight of women and children, and testing for anemia, malaria and Vitamin A deficiency [20, 21, 25]. In this study we only considered women whose blood sample had been drawn for testing, who had a test result for the anemia level, and who were usual members of the household in which they were surveyed. These criteria resulted in 10,956 weighted cases of women age 15–49 years for the three survey years. The total sample included 2672, 2539 and 5745 women in 2006, 2011 and 2016 survey years respectively.

### Measurement of outcome variable

The dependent variable—woman’s anemia status—was recoded from the anemia level variable in the DHS datasets. Anemia level was determined from the result of hemoglobin level from blood testing. During the survey, blood specimens were collected for eligible women who voluntarily consented to be tested. This was done by obtaining a blood sample from a drop of blood taken from a finger prick. Hemoglobin analysis was carried out on site using a portable Hemocue analyser. Results were provided both verbally and in writing and all severe cases were referred for follow-up care. Anemia is marked by low levels of hemoglobin in the blood. For the analysis, all nonpregnant women age 15–49 who had less than 11.0 g of hemoglobin per deciliter (g/dl) were coded as anemic. Among pregnant women, those with hemoglobin values less than 12.0 g per deciliter were considered anemic. Nonpregnant women with hemoglobin values below 4.0 g per deciliter and those above 18.0 g per deciliter (g/dl) were regarded as implausible. Also, hemoglobin values below 3.0 g per deciliter and those above 17.0 g per deciliter (g/dl) in pregnant women were regarded as implausible. All implausible cases were excluded from the analysis. For the analysis, woman’s anemia status was recoded into a binary outcome variable. All women whose anemia level was severe, moderate, or mild, were recoded as yes, and nonanemic cases were recoded as no.

### Measurement of other explanatory variables

For the analysis, covariates were selected based on literature [4, 26, 27]. Covariates were grouped into three categories: community, household and individual variables. Community-level variables included place of residence and region. The region variable for the 2011 and 2016 UDHS was recoded as in the 2006 UDHS, for comparability. It was categorized as Kampala, Central 1, Central 2, East Central, Eastern, Northern, West Nile, Western, and South Western.

Household-level variables included wealth index, sex of the household head, type of toilet facility, source of drinking water, and number of children in the household. Wealth index is a composite measure of a household’s living standards. It is calculated using data on a household’s ownership of assets, household construction materials, and water and sanitation facilities [28]. Wealth index was coded as 1 poorest, 2 poorer, 3 middle, 4 richer, and 5 richest. Sex of the household head was coded as 1 male and 2 female. Type of toilet facility was recoded as 1 improved toilet, 2 shared toilet, 3 nonimproved toilet, and 4 no facility. Source of drinking water was grouped into improved and nonimproved as in the DHS grouping.

Individual-level variables for women included age, educational attainment, involvement in decision-making, ever giving birth, access to health services and pregnancy status. Age was coded into seven 5-year groups: 15–19, 20–24, 25–29, 30–34, 35–39, 40–44, and 45–49, for better illustration of results [20]. Educational attainment was coded as 0 no education, 1 primary, 2 secondary, and 3 higher. Involvement in decision-making was generated from three variables: Women who were involved in decision-making individually or jointly with their partner regarding spending of their income, their own health care, and household purchases were recoded as 1 involved, otherwise 0 not involved. All missing cases were recoded as 9.

Women who reported having given birth were recoded as 1 yes, otherwise 0 no. Access to health care was recoded as 1 yes, otherwise 0 no, depending on whether distance to facility was reported as a big problem in accessing health care or not. Pregnancy status was coded as 1 yes for women who reported that they were pregnant at the time of the survey, and 0 no for women who were not pregnant or not sure of their status.

### Statistical analyses

Only women who had plausible results of the blood hemoglobin levels were included in the analyses. Data were weighted using the women’s individual sample weight to adjust for nonresponse and disproportionate selection. The *svy* command was used to account for complex survey design. The independent variables were tested for multicollinearity using the pairwise correlation coefficient and only variables with a relationship below 0.5 cutoff were included in the analysis [29].

Bivariate analyses were conducted to examine association between the dependent variable (anemia) and the explanatory variables. Pearson’s chi-squared (χ2) tests were used to examine the significant differences between anemia and the explanatory variables. Statistical significance using *p*-values was set at *p* < 0.05. Multivariate logistic regressions were used to examine the relationship between anemia status and the determinant variables. The results are presented for four models: Model 1 for 2006; Model 2 for 2011; Model 3 for 2016; and Model 4 for pooled data for the 3 survey years. Adjusted odds ratios and 95% confidence intervals are presented. All analyses were conducted using Stata version 15, and results are reported for the UDHS survey years 2006, 2011, and 2016.

## Results

### Descriptive and bivariate analyses

Results of the analysis of data presented in Table 1 show that 64, 31, and 38% of pregnant women were anemic in 2006, 2011, and 2016 respectively, substantially higher than the prevalence of anemia among nonpregnant women, at 48, 22, and 31% in the respective survey years. By women’s involvement in household decision-making, 52, 24, and 30% of women who reported being involved in decision-making in 2006, 2011, and 2016 respectively were anemic, somewhat lower than the 53, 30, and 36% of women who were not involved in decision-making. Anemia was also associated with household toilet facility; across the survey years 59, 33 and 39% of women in households that reported no toilet facility were anemic. Region was significantly associated with anemia among women, though the prevalence varied across regions. Further, women in poorer households had higher prevalence of anemia than those in wealthier households across the survey years.

**Table 1:** Proportion of women age 15–49 who were anemic over the survey years by background characteristics, UDHS 2006, 2011, 2016

Characteristic	2006 UDHS		2011 UDHS		2016 UDHS		Pooled Data	
	p-value/ percent	*n*	p-value/ percent	*n*	p-value/ percent	*n*	p-value/ percent	*n*
Place of residence	(0.000)		(0.140)		(0.003)		(0.000)	
Urban	35.0	429	19.9	509	27.5	1485	27.2	2423
Rural	52.5	2244	23.8	2030	32.9	4260	35.9	8534
Region	(0.000)		(0.000)		(0.000)		(0.000)	
Kampala	33.2	204	19.6	243	25.3	294	25.6	741
Central1	59.7	284	23.6	261	27.8	757	33.9	1303
Central2	42.1	240	30.3	243	31.6	614	33.6	1097
East Central	48.9	257	29.8	269	39.2	608	39.1	1134
Eastern	50.4	369	28.4	378	27.3	916	32.7	1663
Northern	63.8	405	21.0	290	41.2	754	43.4	1449
West Nile	37.4	149	32.1	161	39.8	374	37.4	685
Western	45.5	409	17.4	372	30.5	754	31.3	1535
South Western	50.1	353	11.7	324	23.0	675	27.4	1352
Wealth index	(0.000)		(0.029)		(0.000)		(0.000)	
Poorest	57.9	468	28.6	446	40.7	1037	42.0	1952
Poorer	55.9	520	26.4	448	32.9	1029	37.4	1998
Middle	50.4	500	19.4	469	30.8	1079	32.9	2048
Richer	48.1	527	22.2	540	31.0	1168	32.9	2235
Richest	39.6	657	20.2	636	25.0	1432	27.4	2725
Type of toilet facility	(0.001)		(0.044)		(0.000)		(0.000)	
Improved toilet	44.8	244	21.7	422	29.7	1318	29.8	1985
Shared toilet	42.2	431	22.9	501	26.8	1090	29.2	2022
Nonimproved toilet	50.7	1701	22.0	1403	33.2	3018	35.5	6122
No facility	58.8	291	33.0	212	39.3	319	44.6	822
Source of drinking water	(0.071)		(0.948)		(0.023)		(0.001)	
Improved	48.1	1849	23.0	1865	30.6	4585	32.8	8298
Nonimproved	53.2	823	23.2	675	35.3	1160	37.8	2659
Sex of the household head	(0.587)		(0.621)		(0.199)		(0.977)	
Male	50.1	1842	23.4	1715	30.8	3677	34.0	7233
Female	48.7	831	22.4	824	32.7	2069	34.0	3724
Age group	(0.295)		(0.008)		(0.113)		(0.021)	
15–19	44.8	578	19.2	603	32.4	1290	29.9	2471
20–24	50.7	518	24.5	443	33.7	1160	36.0	2122
25–29	50.6	458	22.1	474	27.2	944	31.6	1875
30–34	50.2	396	18.1	323	30.8	802	33.1	1564
35–39	54.4	308	30.8	319	30.6	645	36.4	1272
40–44	51.1	240	25.9	195	33.5	538	36.3	973
45–49	48.9	174	27.1	181	32.9	366	35.3	722
Woman’s level of education	(0.018)		(0.211)		(0.050)		(0.000)	
No education	56.0	532	27.5	308	37.2	567	42.2	1406
Primary	49.2	1585	22.9	1537	31.6	3308	33.8	6431
Secondary	45.7	461	22.5	563	30.1	1460	31.3	2485
Higher	41.4	94	16.8	131	28.6	409	28.0	635
Woman is currently pregnant	(0.000)		(0.006)		(0.006)		(0.000)	
No	47.6	2344	22.1	2257	30.9	5156	32.9	9756
Yes	64.4	329	30.6	283	37.3	589	43.2	1201
Woman has ever given birth	(0.001)		(0.017)		(0.750)		(0.001)	
No	43.2	608	19.3	658	31.1	1446	30.9	2712
Yes	51.6	2064	24.4	1881	31.7	4299	35.0	8245
Whether woman is involved in decision-making	(0.008)		(0.019)		(0.085)		(0.000)	
No	52.6	377	29.6	310	35.7	437	39.7	1124
Yes	51.9	1369	23.9	1278	30.3	3107	34.0	5755
Missing	45.2	926	19.9	951	32.4	2201	32.4	4078
Distance to facility a big problem to woman	(0.182)		(0.127)		(0.019)		(0.000)	
No	48.0	1252	21.7	1466	30.3	3581	31.8	6299
Yes	51.1	1418	25.0	1073	33.6	2164	36.9	4656
Total	49.7 (N = 2672)		23.1 (N = 2539)		31.5 (N = 5745)		34.0 (N = 10,957)	

### Multivariate logistic regression analysis

Table 2 shows that women who were pregnant at the time of the survey had higher odds of being anemic compared with those who were not pregnant, for all the survey years (OR 2.00, 95% CI 1.49–2.67; OR 1.47, 95% CI 1.02–2.10; OR 1.33, 95% CI 1.07–1.65) and for the pooled results (OR 1.49, 95% CI 1.28–1.73). Higher odds for anemia were observed among women with nonimproved sources of drinking water in 2016, and for the pooled results (OR 1.32, 95% CI 1.08–1.61; OR 1.18, 95% CI 1.03–1.36) compared with women in households with improved sources of water. Also, women age 35–39 had higher odds for anemia compared with women age 15–19 in 2011 (OR 1.99, 95% CI 1.27–3.14). Rural women had higher odds (OR 1.61, 95% CI 1.01–2.56) of anemia compared with urban women in 2006.

**Table 2:** Adjusted odds ratios (AORs) for anemia among women age 15–49 across the survey years, UDHS 2006, 2011, 2016

Characteristic	2006 UDHS		2011 UDHS		2016 UDHS		Pooled Data	
	Odds Ratio	[95% CI]	Odds Ratio	[95% CI]	Odds Ratio	[95% CI]	Odds Ratio	[95% CI]
Place of residence (rc: Urban)								
Rural	1.61*	1.01–2.56	1.13	0.71–1.80	0.94	0.77–1.15	1.10	0.92–1.32
Region (rc: Kampala)								
Central1	1.60	0.78–3.29	0.95	0.49–1.85	0.92	0.63–1.32	1.14	0.85–1.54
Central2	0.76	0.38–1.52	1.40	0.76–2.55	1.00	0.67–1.48	1.07	0.79–1.45
East Central	0.98	0.49–1.95	1.35	0.74–2.46	1.40	0.97–2.03	1.33	0.98–1.80
Eastern	0.93	0.47–1.84	1.10	0.55–2.18	0.78	0.54–1.13	0.91	0.67–1.24
Northern	1.65	0.81–3.36	0.66	0.34–1.29	1.35	0.92–1.98	1.30	0.95–1.78
West Nile	0.58	0.30–1.13	1.32	0.69–2.54	1.31	0.83–2.05	1.15	0.81–1.61
Western	0.79	0.39–1.60	0.67	0.32–1.40	0.92	0.61–1.37	0.87	0.63–1.21
South Western	1.02	0.49–2.12	0.40*	0.19–0.84	0.59*	0.38–0.92	0.71*	0.51–1.00
Wealth index (rc: Poorest)								
Poorer	1.06	0.78–1.45	0.93	0.64–1.35	0.80*	0.64–0.99	0.86	0.74–1.01
Middle	0.92	0.64–1.31	0.74	0.48–1.15	0.74*	0.58–0.95	0.76**	0.63–0.91
Richer	0.84	0.60–1.19	0.85	0.56–1.29	0.74*	0.57–0.97	0.75**	0.62–0.90
Richest	0.71	0.47–1.08	0.77	0.39–1.53	0.55***	0.40–0.76	0.59***	0.47–0.76
Type of toilet facility (rc: Improved)								
Shared toilet	0.89	0.62–1.28	1.11	0.69–1.78	0.89	0.69–1.14	0.93	0.77–1.12
Nonimproved toilet	1.01	0.70–1.46	1.00	0.68–1.49	1.00	0.81–1.24	0.98	0.83–1.16
No facility	1.03	0.64–1.68	1.45	0.82–2.57	0.90	0.65–1.25	1.01	0.80–1.29
Source of drinking water (rc: Improved)								
Nonimproved	1.09	0.85–1.40	1.16	0.86–1.56	1.32**	1.08–1.61	1.18*	1.03–1.36
Sex of the household head (rc: Male)								
Female	1.01	0.80–1.28	0.96	0.74–1.25	1.05	0.90–1.22	1.03	0.92–1.15
Age of woman (rc: 15–19)								
20–24	1.01	0.70–1.47	1.32	0.85–2.05	1.01	0.80–1.28	1.06	0.88–1.26
25–29	0.99	0.66–1.46	1.16	0.75–1.80	0.75*	0.57–0.99	0.88	0.72–1.07
30–34	0.95	0.65–1.39	0.93	0.57–1.52	0.88	0.64–1.20	0.91	0.73–1.12
35–39	1.19	0.77–1.84	1.99**	1.27–3.14	0.86	0.64–1.17	1.11	0.89–1.37
40–44	1.05	0.66–1.66	1.48	0.87–2.52	0.98	0.72–1.34	1.07	0.85–1.34
45–49	0.92	0.58–1.46	1.68	0.96–2.95	1.01	0.73–1.40	1.07	0.84–1.36
Woman’s level of education (rc: None)								
Primary	0.89	0.71–1.12	0.89	0.64–1.25	0.85	0.69–1.04	0.88	0.76–1.01
Secondary	1.13	0.81–1.58	1.07	0.64–1.78	0.96	0.74–1.25	1.02	0.84–1.23
Higher	0.94	0.56–1.57	0.81	0.40–1.62	1.07	0.73–1.59	1.01	0.76–1.35
Woman currently pregnant (rc: No)								
Yes	2.00***	1.49–2.67	1.47*	1.02–2.10	1.33*	1.07–1.65	1.49***	1.28–1.73
Woman has ever given birth (rc: No)								
Yes	1.34	0.96–1.88	0.90	0.59–1.35	1.20	0.93–1.54	1.17	0.98–1.40
Woman involved in decision-making (rc: No)								
Yes	1.03	0.79–1.34	0.76	0.51–1.15	0.84	0.66–1.08	0.86	0.73–1.02
Missing	1.04	0.76–1.44	0.72	0.46–1.14	1.00	0.75–1.32	0.93	0.77–1.13
Distance to facility a problem (rc: No)								
Yes	0.92	0.76–1.12	1.12	0.88–1.43	1.01	0.88–1.16	1.02	0.92–1.13
Survey year (rc: 2006)								
2011							0.30***	0.26–0.36
2016							0.47***	0.41–0.54
Total observations	2645		2580		5799		11,024	

Level of significance *** *p* < 0.001, ** *p* < 0.01, * p < 0.05

CI: Confidence interval

rc: Reference category

Compared with the poorest households, the odds of anemia in women in the wealthier household quintiles were lower in 2016 (OR 0.80, 95% CI 0.64–0.99; OR 0.74, 95% CI 0.58–0.95; OR 0.74, 95% CI 0.57–0.97; OR 0.55, 95% CI 0.40–0.76) and for the pooled results (OR 0.76, 95% CI 0.63–0.91; OR 0.75, 95% CI 0.62–0.90; OR 0.59, 95% CI 0.47–0.76). The same pattern was observed in 2006 and 2011, although it was not statistically significant. Women age 25–29 had lower odds for anemia compared with women age 15–19 in 2016 (OR 0.75, 95% CI 0.57–0.99). Women in the South Western region had lower odds for anemia compared with women in Kampala in 2011 (OR 0.40, 95% CI 0.19–0.84) and 2016 (OR 0.59, 95% CI 0.38–0.92), and for the pooled results (OR 0.71, 95% CI 0.51–1.00). The pooled results in Table 2 show lower odds for women anemia in the 2011 and 2016 surveys (OR 0.30, 95% CI 0.26–0.36; OR 0.47, 95% CI 0.41–0.54) compared with 2006.

## Discussion

This paper examined the trends and determinants of anemia among women of reproductive age in Uganda. Despite the many frameworks and policies for addressing Uganda’s nutrition issues [16, 30, 31], the country still faces various nutrition challenges.

This study shows that, although the prevalence of anemia among women in Uganda has declined since the 2006 UDHS, it increased between 2011 and 2016 and still affects a sizable proportion of women of reproductive age. The overall prevalence of anemia among women in this study was 50% in 2006, 23% in 2011, and 32% in 2016. Common risk factors for anemia among women include pregnancy, source of drinking water, age, residence, household wealth, and region.

Results show that the prevalence of anemia in women of reproductive age in Uganda was 32%, nearly the same as the global average of 33% in 2016 [4]. This rate remains high despite the interventions by the Ministry of Health, including the indoor residual spraying, distribution of free insecticide-treated mosquito nets, and iron and vitamin supplementation for pregnant women, among others [16]. Poor health is a hindrance towards achieving the ultimate goal of health for all the population. This implies that substantial effort is needed to reduce the prevalence of anemia among women, since it affects maternal and child health and other development outcomes.

Pregnancy increased the odds of anemia in women. In pregnancy there is blood volume expansion [32], which consequently increases iron and folic acid demand. More blood is produced to support the growth of the baby but this is only possible if the body is able to produce the required amount of red blood cells. These physiological changes increase the risk of anemia, especially if the woman’s dietary needs are not met. Anemia in pregnant women leads to poor fetal outcomes such as low birth weight and stillbirths, and can also lead to death of the mother [10, 19]. More effort is therefore needed to emphasize proper nutrition for women during pregnancy and breastfeeding. Intermittent preventive treatment doses of sulphadoxine-pyrimethamine should also be given to pregnant women, including those in rural areas, to prevent malaria, since this is a predisposing factor for anemia [33, 34].

Our hypothesis related to residence and anemia was partially supported. Rural women were more likely to be anemic than urban women. Higher prevalence of anemia in rural areas may be attributed to problems of access to services including health services and household poverty. There is a considerably higher proportion of rural women who belong to poor households, that may have challenges with access to proper nutrition. This is consistent with findings elsewhere that showed that rural residence is related to poor health outcomes [5, 35] and lack of access to services including education and nutrition [36]. Though rural households primarily depend on agriculture, and therefore may have access to foods and vegetables, they are more likely to suffer from non-iron deficiency anemia. This may be caused by infections, chronic diseases or zinc deficiency.

We further hypothesized that women in poor households are more likely to be anemic. This hypothesis is partially supported by the results of this study. Similar to findings elsewhere, women in the poorest households had more anemia cases compared with women in wealthier households [37, 38]. This could be explained by the inability of women in poorer households to afford a good diet [39], pay for health care [40], and practice good sanitation compared with women in wealthier households. Poor households tend to consume foods that are nutrient-poor, and are likely to be food-insecure. This implies that members in such poor households are at increased risk of anemia. Poor women are further likely to have challenges with regards to autonomy in household decision making. Women disproportionately bear the burden of provision of basic needs for their household members [41]. This implies that household poverty is likely to impact access and use of services by these women. There is need to ensure that all women have access to health services and information on proper feeding [42], especially in rural areas. In addition, there is need to strengthen poverty alleviation programs and women’s employment opportunities in order to improve their economic status. There is also need to boost agricultural innovations and investments since a sizable proportion of households depend on agricultural livelihoods [43]. This has the potential not only to increase incomes of women, but also increase food and nutrition security among households. As women gain access to income, they spend more on health, nutrition and education within the household.

Though Humphrey et al. in 2009 [44], in a study in rural Zimbabwe, reported that water, sanitation, and hygiene interventions are unlikely to reduce stunting or anemia in children, our study found that source of drinking water was significantly associated with anemia in women. Results showed more anemia cases among women who used unsafe water compared with those with safe sources. This points to the risk of waterborne diseases such as diarrhea, dysentery, and typhoid, which may increase the risk of anemia in households that draw water from nonimproved sources. Waterborne diseases may be caused due to exposure to fecal matter in such open sources of water. There is therefore need for the government to ensure that all households have access to safe water sources, so that such diseases are prevented. Prevention of these diseases is important to the particular households and also reduces the circulation of diseases within the community.

Women’s age was associated with anemia prevalence. Higher levels of anemia were observed among older women. This may be explained by blood loss during childbirth, which increases the risk of anemia [6]. On the other hand, anemia among teenagers may be explained by the increased needs for iron during adolescence and blood loss during menstruation cycles [45, 46]. Family planning interventions should be strengthened to prevent early pregnancy through reaching out to adolescents in school and also those out of school. Further, all women of reproductive age should take iron and folic acid supplementation to prevent anemia [47]. Multisectoral programmes aimed at addressing nutrition-related problems among all socioeconomic categories of women would be important in reducing anemia in women.

The research findings should be considered in light of some limitations with the study. The study was based on data from a cross-sectional survey, and therefore it was not possible to assess the cause-effect relationship between variables. It is self-reported information provided by survey respondents, which may be subject to recall bias. The study did not include dietary data because this information was only collected for children under age 24 months, yet, the study considered women of the reproductive age 15–49 years. It was therefore not possible to assess associations between diet and the anemia status of women in our sample. Nevertheless, the study offers insights into the high levels of anemia among women of reproductive age in Uganda, that could be a basis for interventions.

## Conclusion

The results suggest that empowerment of women with economic resources, power to make decisions on spending household incomes, and access to health care should be prioritized in order to reduce anemia in women. More focus should be given to nutrition during antenatal and postpartum visits to health facilities. Health facilities should encourage supplementary feeding for boosting immunity for pregnant mothers since pregnancy increases the risk of anemia. This can be supported by setting up nutrition units in health facilities. Teenagers need to be continuously sensitized to the risks of teenage pregnancies and supported to stay in school. In cases of pregnancies, adolescent-friendly services should be available to support teenagers during pregnancy and motherhood. Family planning services should be extended to all women and teenagers who need them. Effective implementation of interventions to combat anemia in women will significantly reduce morbidity and mortality and other adverse consequences of anemia.

## Data Availability

The datasets analyzed during the current study are available in the public domain at (URL: https://www.dhsprogram.com/data/available-datasets.cfm) upon registration at the DHS program website. We used the UGIR files (Women’s recode – Women age 15–49 with complete interviews).

## Abbreviations

CI: Confidence Interval
UBOS: Uganda Bureau of Statistics
UDHS: Uganda Demographic and Health Surveys
